# A cognitive screening program in community‐based medical clinics to facilitate Latino participation in Alzheimer's disease research

**DOI:** 10.1002/alz.71132

**Published:** 2026-01-22

**Authors:** David P. Salmon, Caytre Ede, Christina Gigliotti, Emily A. Little, Melanie L. Quiring, Roberto Gratianne, Jairo A. Romero, Diane M. Jacobs, Guerry M. Peavy, Douglas Galasko

**Affiliations:** ^1^ Department of Neurosciences University of California San Diego USA; ^2^ Shiley‐Marcos Alzheimer's Disease Research Center University of California San Diego USA; ^3^ Department of Gerontology University of Southern California Los Angeles USA; ^4^ South Bay Neurology Group Chula Vista California USA; ^5^ Department of Medicine University of California San Diego USA

**Keywords:** ADRD research, cognitive screening, community‐based research, Latino, recruitment

## Abstract

**INTRODUCTION:**

Relatively low rates of Latino participation in Alzheimer's disease and related dementias (ADRD) research makes it difficult to determine whether identified disease mechanisms, risk factors, or novel treatments generalize to this population.

**METHODS:**

We introduced ADRD research opportunities through a model cognitive screening program in primary care and neurology specialty care clinics in areas with high proportions of Latino residents.

**RESULTS:**

Out of 523 Latino adults (mean age = 72.1 years, mean education = 6.8 years, 62.1% female, 88.1% tested in Spanish), 520 allowed the use of screening data for research, and high percentages agreed to a research registry with contact about ADRD research opportunities (primary care: 91.9%, neurology: 86.6%). Registrants supported 368 referrals to 45 studies (21% successfully recruited). Thirty‐one individuals participated in ≥1 study, producing 79 enrollments across 15 ADRD studies.

**DISCUSSION:**

Results demonstrate that combining a needed clinical service with recruitment efforts can enhance participation of older Latino adults in ADRD research.

## Highlights

- Older Latinos (>99%) allowed the use of cognitive screening data for research purposes.
- Primary care (91%) and neurology (87%) patients allowed ADRD research solicitation.
- Twenty‐one percent of referrals of those agreeing to contact (79/368) led to research recruitment.
- Thirty‐one participants accounted for 79 distinct enrollments across 15 ADRD studies.
- Combining clinical service with recruitment enhances Latino research participation.

## 1 BACKGROUND

The prevalence of Alzheimer's disease and related dementias (ADRD) in the United States may be as much as 1.5 times higher in Latinos than in non‐Latino Whites,^1^, ^2^, ^3^, ^4^ yet the rate of Latino participation in ADRD research remains low.^5^, ^6^ This reduced representation makes it difficult to determine whether or not newly identified disease mechanisms, risk factors, or novel treatments for ADRD generalize to this population. Barriers to Latino participation in research have been identified, including low awareness of Alzheimer's disease (AD) in the community, few research opportunities, transportation limitations, financial burden (e.g., time off work, gas, parking), and general distrust of the research process.^7^, ^8^, ^9^, ^10^, ^11^ Additional barriers arising from the researchers’ side are lack of culturally and linguistically appropriate tests with normative data for reliable cognitive assessment of older Latino adults,^11^, ^12^, ^13^ a shortage of Spanish‐speaking clinicians and staff at study sites, costs associated with the translation of consent documents and recruitment materials,^14^ and restrictive study inclusion and exclusion criteria that do not account for the unique characteristics of the population (e.g., non‐English speaking, low education, increased vascular disease).

Overcoming many of these barriers can be facilitated by the application of community‐based participatory research principles.^15^ These principles advocate building on the strengths and resources of the community, addressing community‐defined problems, engaging in research that benefits both science and the community, and establishing a long‐term commitment through partnerships and dissemination of acquired knowledge.^11^, ^15^ We applied this approach by adapting a model cognitive screening program we developed for the primary care setting^16^ and placing it in primary care and neurology specialty care clinics located in areas of San Diego County with a high proportion of Latino residents. The cognitive screening program provided a brief objective assessment of memory and other cognitive abilities in those patients where the primary care provider or neurologist suspected but was unsure of the presence of cognitive deficits, and offered an opportunity to introduce patients to the topic of ADRD research and describe research opportunities in projects affiliated with the University of California San Diego (UCSD) Shiley–Marcos Alzheimer's Disease Research Center (ADRC).

The purpose of this study was to evaluate the effectiveness of this approach for recruiting older Latino adults with suspected cognitive decline into ADRD research activities and to compare the characteristics of those screened and recruited through primary care or neurology specialty care clinics. We hypothesized that placing the discussion of research within the context of trusted community‐based medical care would lead to a high rate of participation and that this would work equally well across primary care and neurology specialty care settings. Participation was examined at three levels: (1) allowing screening data to be used for research purposes, (2) recruitment into a research registry for learning about and possibly participating in specific ADRD research studies, and (3) recruitment from the registry into an active ADRD research study. We further hypothesized that those recruited from primary care or neurology specialty care would share demographic and clinical features, but those from neurology specialty care would be more likely to have objective cognitive impairment not explained by secondary factors (e.g., depression, medications, medical conditions) since they were presumably vetted for these factors at the primary care level before being referred to a specialist.

## 2 METHODS

### 2.1 Participants

Cognitive screening referrals were generated from clinics at the San Ysidro Health Center and the South Bay Neurological Group, which are both located in a southern region of San Diego County, California, which borders Mexico and has a high proportion of Latino residents (i.e., 63% of a population > 300,000 according to the 2020 U.S. Census). The San Ysidro Health Center is a non‐profit healthcare organization with 3000 staff members who provide culturally proficient health care to over 145,000 patients through an integrated network of medical clinics. Comprehensive primary care services for older adults are provided through the organization's Department of Family Medicine. Referrals for cognitive screening came from 14 general practice physicians in family medicine, five physicians in internal medicine, one family medicine physician assistant, and one family medicine nurse practitioner. The South Bay Neurological Group is a neurology medical practice in Chula Vista, CA, that specializes in the diagnosis and treatment of a wide variety of neurological disorders, including stroke, vascular dementia, and AD. The group works with other treating healthcare providers to provide integrated care to a patient base that is largely Mexican American. A Spanish‐English bilingual neurologist with Latino/Hispanic heritage was the primary source of referrals for cognitive screening at this site.

**RESEARCH IN CONTEXT**

1. **Systematic review**: The authors reviewed the literature on methods used to recruit older Latino adults into ADRD research using traditional sources (e.g., PubMed). Little information was available on the effectiveness of using cognitive screening to introduce ADRD research opportunities into community‐based primary care and specialty neurology clinics that serve this vulnerable population.
2. **Interpretation**: Our findings showed that a high percentage of older Latino adults allowed cognitive screening data to be used for research purposes and agreed to be contacted about specific ADRD research opportunities. Approximately 21% of referrals among those who agreed to be contacted led to successful recruitment, with 7% of individuals who were screened participating in one or more ADRD studies.
3. **Future directions**: Additional research is needed to identify barriers to Latino participation in ADRD research and how they might be overcome by community‐based, pragmatic approaches that incorporate clinical care.

**FIGURE 1:**
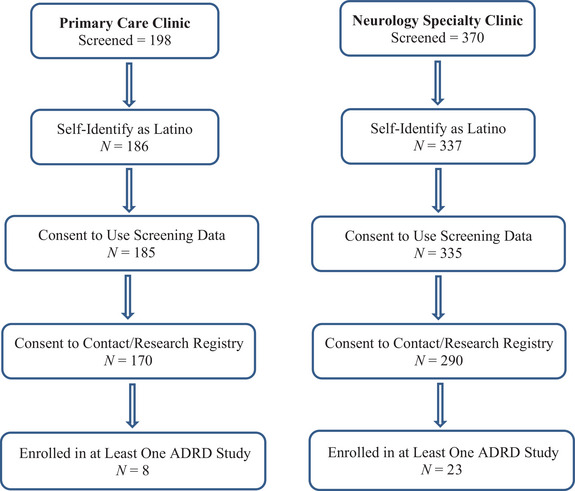
Flow chart showing number of patients screened in each clinic setting who self‐identified as Latino, allowed their screening data to be used for research purposes, consented to subsequent contact about Alzheimer's disease and related dementias (ADRD) research participation and listing in the ADRC Research Registry, and subsequently enrolled in at least one ADRD research study.

Guidelines for referral to cognitive screening were ≥60 years of age, a complaint of decline in memory or other aspect of cognition from the patient, a family member, a knowledgeable informant, or physician who suspects memory decline, and uncertainty by the physician that the complaint reflects a true cognitive deficit (i.e., patients with frank, moderate to severe dementia should not need screening). A total of 568 patients were screened, with 523 self‐identified as Latino or Hispanic (92.1%) (Figure 1). Written informed consent to use the screening results in a research study on the efficacy and utilization of memory screening in the clinic setting was provided by 520 of these Latino patients, 185 who were screened at the San Ysidro Health site and 335 who were screened at the South Bay Neurological Group site. The majority of patients (86.2%) were tested in Spanish at their request.

### 2.2 Cognitive screening procedures

Participants were scheduled by their healthcare provider and tested individually in their preferred language (Spanish or English) by a highly proficient Spanish‐English bilingual psychometrist from the UCSD ADRC. Testing took place in a quiet room at one of the two clinic locations. A brief questionnaire was administered first that asked about demographic information (age, education, sex), history of head injury (yes/no), history of stroke (yes/no), current or history of depression diagnosis or treatment (yes/no), and current major medical illnesses and medications. Education grade equivalency was measured by the Woodcock‐Munoz Language Survey Letter‐Word Identification Test.^17^ Subjective cognitive decline (SCD) was assessed with five yes/no questions about current (1) persistent memory difficulties, (2) difficulty finding words, (3) difficulty remembering people's names, (4) misplacing belongings, and (5) difficulty completing complex tasks. A total SCD score (range 0 to 5) was created by assigning each positive response one point and summing across the five questions. Cognitive testing was carried out using standardized neuropsychological tests that included the Mini‐Mental State Examination (MMSE),^18^ Wechsler Memory Scale‐Revised Logical Memory Test (immediate and delayed recall of Story A only),^19^ Consortium to Establish a Registry for Alzheimer's Disease (CERAD) Word List Learning Task,^20^ Category Fluency Test (“animals”), Clock Drawing Test, and Trail‐Making Test Parts A and B (TMT‐A and TMT‐B).^21^ All tests had been translated into Spanish by the UCSD ADRC.^22^ A self‐reported short (15‐item) version of the Geriatric Depression Scale (GDS)^23^ was completed. All screening procedures required approximately 40 min.

The psychometrist conveyed the cognitive test scores, GDS score, and summary clinical information to off‐site neuropsychologists and behavioral neurologists with expertise in ADRD for review. Test scores were classified as normal or impaired compared to age‐referenced normative data from Latino participants in the ADRC cohort study.^22^ Age‐ and education‐adjusted standard scores of −1.5 or more below the normative mean for a given test were considered impaired. A cognitive domain (e.g., memory, language, executive function) was considered impaired if scores on one or more test measures within that domain were impaired unrelated to extraneous factors (e.g., illiteracy, extremely low education, compromised vision or hearing) in the judgement of the reviewing experts.

When the review was complete, the MMSE score and the experts’ finding of presence or absence of cognitive impairment and possible contributory factors (e.g., depression) was returned to the referring physician within 1 to 2 weeks using one of the following template statements (sometimes with slight modification):

1. Mild memory impairment with good performance in other cognitive domains suggestive of mild cognitive impairment (MCI): A comprehensive assessment with further medical work‐up and follow‐up and/or referral to a specialist should be considered. MCI can be a prodromal stage of AD but also may result from conditions such as head trauma, stroke, alcohol, depression, or general medical illness. In people with low levels of education, the diagnosis of MCI requires caution.
2. No significant memory impairment, but mild impairment in other cognitive domain(s) suggestive of MCI: A comprehensive assessment with further medical work‐up and follow‐up and/or referral to a specialist should be considered. MCI can be a prodromal stage of AD but also may result from conditions such as head trauma, stroke, alcohol, depression, or general medical illness. In people with low levels of education, the diagnosis of MCI requires caution.
3. Mild memory impairment with mild impairment in other cognitive domain(s): This may indicate early dementia. We recommend that the patient undergo a comprehensive assessment to confirm these results and determine the likely underlying causes. Note that patients with significant cognitive defects need to be reported to the Department of Motor Vehicles (DMV). The cognitive screening results should be reviewed, and if they are consistent with a clinical impression of sufficient cognitive decline to affect driving, we recommend reporting the patient to the DMV.
4. MCI and symptoms of depression on the GDS: We suggest considering further work‐up and treatment for depression and/or referral to a specialist. Vascular risk factors (e.g., hypertension, diabetes, increased cholesterol) or uncontrolled general medical problems should also be considered.
5. No evidence of significant impairment of memory or other areas of cognition, but symptoms of depression on the GDS: We suggest considering further work‐up and treatment for depression and/or referral to a specialist.
6. No evidence of significant impairment of memory or other areas of cognition – the patient's symptoms most likely reflect brain changes accompanying normal aging. Re‐evaluation should occur in one year or sooner if cognitive symptoms worsen.

This information could then be used to assist the physician in developing a diagnosis and plan of care based on their overall knowledge of the patient's medical history, other test results, and unique medical status. This screening approach was shown to be highly effective and well utilized by physicians in previous work.^16^

### 2.3 Presentation of ADRD research opportunities

After completing the cognitive screening procedures, the psychometrist presented a brief overview of ADRC‐affiliated research activities and asked the patient if they could be contacted about participation in ADRD research studies. They were asked to provide written informed consent to be listed in an ADRC research registry that would include their demographic and contact information so they could be matched with specific research projects and subsequently contacted about participation. They were told that if they agreed to contact and satisfied a particular study's entry criteria, the interested investigator would directly contact them with additional details about the rationale, procedures, and requirements of the particular study and ask if they would participate. They were assured they were free to agree or decline to participate in any study. They were told that they may be contacted by multiple studies. The psychometrist explained that they could remove themselves from the registry at any time and reassured them that their participation in research was entirely voluntary and unrelated to their clinical care.

### 2.4 Informed consent

Cognitive screening was carried out as a research project of the UCSD ADRC and California Alzheimer's Disease Center (CADC) and was free of charge to the patient and healthcare provider. Screening was not contingent upon agreeing to research, but written informed consent to utilize the data for research purposes was obtained from patients, consistent with California state law, following the screening procedures. Deidentified data were included in analyses only if written informed consent to use them was obtained. Written consent was also obtained to include the participant's name, demographic information, screening results, and contact information in a research registry and for permission to contact them about future ADRD research opportunities. The research protocol was reviewed and approved by the human subjects review board at UCSD.

### 2.5 Recruitment through ADRC research registry

The cognitive screening program operated from 2009 to 2019. Recruitment from the research registry that occurred from May 2011 to February 2023 is reported. During that time, 45 unique ADRD studies were recruiting, including the ADRC longitudinal observational study, which involved annual clinical and neuropsychological assessment, AD biomarker acquisition through blood draw and/or lumbar puncture, and neuroimaging. The registry was managed by ADRC personnel who reviewed and fulfilled referral requests from ADRC‐affiliated investigators. Each study specified inclusion and exclusion criteria for those they wished to recruit, and registry participants meeting those criteria were identified and their contact information provided to the study investigator. These potential participants were further vetted by the study investigator, who then contacted those who were study‐appropriate.

### 2.6 Statistical analysis

To determine whether placing the discussion of ADRD research participation within the context of trusted community‐based medical care would lead to a high rate of engagement, we calculated the percentage of those screened who agreed to (1) allow their screening data to be used for research purposes, (2) to be listed in the research registry and contacted about ADRD research opportunities, and 3) participate in an active ADRD research study. To determine whether this approach worked equally well across primary care and neurology specialty care settings, chi‐squared (χ^2^) tests compared the percentage of participants from each clinic that gave permission to be listed in the research registry and the percentage subsequently recruited from the research registry to participate in an ADRC‐affiliated research study (including the ADRC longitudinal observational study). To determine whether those screened in the two settings shared demographic and clinical features, means and standard deviations (SDs) were calculated by setting for age, years of education, grade equivalency, GDS score, SCD score, MMSE score, and all neuropsychological test scores. Age, years of education, grade equivalency, GDS score, and sex distribution were compared across the two clinic settings using *t*‐tests and χ^2^ tests. Scores on the SCD scale, MMSE, and all neuropsychological tests were compared across clinic settings using multiple regression analyses that controlled for age, years of formal education, and sex. Unstandardized beta (*B*) for clinic setting is reported along with the overall significance of the model. Chi‐squared tests were used to compare the proportion of individuals from each clinic classified by screening as normal cognition, depression (without significant cognitive impairment), MCI, or dementia. Multiple logistic regression with forward selection was used to determine whether age, years of education, sex, language of testing, clinic setting, or final screening classification predicted consent to be listed in the research registry. Finally, we calculated the number and percentage of those in the registry who were referred to at least one active ADRD research study and the total number of referrals that were made. The percentages of referrals that lead to successful enrollment and reasons for non‐enrollment were examined. Age, years of education, sex, language of testing, and clinic setting of those successfully enrolled into an ADRD research study were compared to the remainder of those in the registry using *t*‐tests and χ^2^ tests. All analyses were performed using SPSS version 29.0.2.0.

## 3 RESULTS

### 3.1 Demographic and clinical characteristics

The overall sample of Latino patients who consented to have their results used for research (*n* = 520) averaged 72.1 years of age (SD = 9.9; range = 40 to 98 years) and had 6.8 years of formal education (SD = 4.3; range = 0 to 20 years), and 62.1% were female. Average education grade equivalency of those tested in Spanish (*n* = 458; 88.1%) was 11.6 years (SD = 5.32; range = 0 to 18 years). Patients screened in the primary care clinic were younger (*t*[518] = −8.83; *p* < 0.001) and had less formal education (*t*[516] = −3.94; *p* < 0.001) than those screened in the neurology clinic; however, the sites did not differ in grade equivalency of those tested in Spanish (*t*[456] = −1.64; *p* = 0.102) (Table 1). The proportion of those screened who were women was higher in the primary care than in the neurology clinic (χ^2^[1] = 5.21; *p* = 0.022). The sites did not differ in the proportion of patients who reported a prior head injury (χ^2^[1] < 0.01; *p* = 0.980), history of a transient ischemic attack (TIA) or stroke (χ^2^[1] = 2.32; *p* = 0.128), or past (χ^2^[1] = 0.13; *p* = 0.720) or current depression (χ^2^[1] = 2.75; *p* = 0.097). The GDS score was higher in those screened in the primary care clinic than in the neurology clinic (*t*[481] = 8.54; *p* < 0.001), but fewer were taking anti‐depressant medication (χ^2^[1] = 6.89; *p* = 0.009). The sites did not differ in the proportion of patients taking neuroleptic (χ^2^[1] = 0.34; *p* = 0.563) or anticholinergic (χ^2^[1] = 0.09; *p* = 0.770) medication. MMSE scores were lower for those screened in the primary care clinic than in the neurology clinic (*B* = 2.31; *p* < 0.001; *R* ^2 ^= 0.219; F[4, 512] = 35.80; *p* < 0.001) and SCD scores were worse (*B* = −0.943; *p* < 0.001; *R* ^2 ^= 0.127; F[4,512] = 18.58; *p* < 0.001), with a higher proportion of those screened in the primary care than neurology clinic endorsing each SCD question (all *p* < 0.001). A higher (though still small) proportion of those screened in the neurology clinic were taking a cholinesterase inhibitor at the time of screening (χ^2^[1] = 16.24; *p* < 0.001).

**TABLE 1:** Mean (SD) age, education, sex distribution and Mini‐Mental State Examination (MMSE), Geriatric Depression Scale (GDS), and Subjective Cognitive Decline (SCD) scores for patients from primary care versus neurology specialty care clinics. The percentage of patients endorsing each medical history, medication, and SCD item is also shown.

Total screened	Primary care (*n* = 185)	Neurology specialty care (*n* = 335)
Age	67.3 (10.2)	74.8 (8.7)^*^
Education (years)	5.8 (3.7)	7.3 (4.5) ^*^
Grade equivalency	11.0 (5.4)	11.9 (5.2)
Sex (%F)	68.6%	58.5%^*^
MMSE	22.4 (5.3)	24.4 (3.9) ^*^
GDS	6.9 (4.4)	3.7 (3.5) ^*^
**Medical history (% yes)**		
Head injury	22.5%	22.5%
Stroke or transient ischemic attack	14.4%	19.8%
Stroke or transient ischemic attack	19.3%	18.0%
Current depression	32.6%	40.1%
SCD total score (max score = 5)	3.4 (1.5)	2.3 (1.6) ^*^
**SCD Items (% yes)**		
Persistent memory difficulties	72.2%	60.1%^*^
Difficulty finding words	67.6%	46.1%^*^
Difficulty remembering names	61.1%	38.0%^*^
Misplaces belongings	81.1%	54.8%^*^
Difficulty with complex tasks	57.5%	32.3%^*^
**Current medications (% yes)**		
Cholinesterase inhibitor	1.2%	11.3%^*^
Antidepressant	22.5%	33.8%^*^
Neuroleptic	1.1%	1.8%
Anticholinergic	2.9%	3.4%

* Significant group difference (*p* < 0.05).

### 3.2 Cognitive screening test scores and interpretation

Patients screened at the primary care clinic scored worse than those screened at the neurology clinic on Logical Memory Test (Story A) Immediate Recall (*B* = 1.37; *p* < 0.001; *R* ^2 ^= 0.124; F[4, 510] = 18.07; *p* < 0.001) and Delayed Recall (*B* = 1.07; *p* < 0.001; *R* ^2 ^= 0.097; F[4, 507] = 13.60; *p* < 0.001), but not Percent Savings (*B* = 6.68; *p* = 0.190; *R* ^2 ^= 0.005; F[4, 502] = 0.65; *p* = 0.629) (Table 2). Primary care patients also scored worse than neurology specialty care patients on CERAD Word List Trials 1‐3 Recall (*B* = 1.90; *p* < 0.001; *R* ^2 ^= 0.192; F[4, 503] = 29.95; *p* < 0.001), Delayed Recall (*B* = 0.894; *p* < 0.001; *R* ^2 ^= 0.195; F[4,502] = 30.39; *p* < 0.001) and Correct Recognition (*B* = 1.20; *p* < 0.001; *R* ^2 ^= 0.071; F[4,500] = 9.62; *p* < 0.001). Patients from primary care scored worse than those from neurology specialty care on the TMT‐A (*B* = −12.13; *p* = 0.001; *R* ^2 ^= 0.248; F[4, 478] = 39.43; *p* < 0.001), but not TMT‐B (*B* = −7.10; *p* = 0.455; *R* ^2 ^= 0.230; F[4, 338] = 25.20; *p* < 0.001); however, TMT‐B was not attempted by more than 30% of all participants due to not understanding test instructions. Primary care patients performed worse than neurology patients on the Clock Drawing Test (*B* = 0.198; *p* = 0.011; *R* ^2 ^= 0.099; F[4,502] = 20.34; *p* < 0.001) and the Category Fluency Test (*B* = 1.55; *p* < 0.001; *R* ^2 ^= 0.248; F[4,504] = 13.92; *p* < 0.001).

**TABLE 2:** Mean (SD) cognitive test scores and the number (and percentage) of patients from primary care versus neurology specialty care clinics assigned to each cognitive screening classification.

	Primary care	Neurology specialty care
Total screened	(*n* = 185)	(*n* = 335)
**Cognitive screening tests**		
Logical memory test (Story A)		
Immediate	6.9 (4.3)	7.8 (4.0)
Delayed	5.3 (4.2)	5.8 (4.2)
Percent savings (%)	65.2 (37.2)	68.9 (54.1)
Trail‐Making Test A (seconds)	87.9 (39.0)	80.0 (40.1)
Trail‐Making Test B (seconds)	230.0 (80.0)	230.3 (84.0)
CERAD word list learning		
Trials 1 to 3 total	13.2 (4.9)	14.1 (4.2)
Delayed recall	3.9 (2.6)	4.2 (2.5)
Correct recognition	17.0 (2.8)	17.9 (2.4)
Clock drawing test	1.8 (0.8)	2.0 (0.8)
Clock drawing test	1.8 (0.8)	2.0 (0.8)
Category fluency (“Animals”)	13.3 (4.6)	14.1 (4.7)
**Cognitive screening classification**		
Normal cognition	23 (12.4%)	77 (23.0%)
Depression	57 (30.8%)	59 (17.6%)
Mild cognitive impairment	27 (14.6%)	90 (26.9%)
Dementia	78 (42.2%)	109 (32.5%)

Abbreviation: CERAD, consortium to establish a registry for alzheimer's disease.

The distributions of participants categorized by screening as normal cognition, depression (with normal cognition), MCI (with or without symptoms of depression), or dementia (with or without symptoms of depression) at the primary care and neurology sites differed significantly (χ^2^[3] = 27.26; *p* < 0.001) (Table 2). A higher percentage of patients from the primary care clinic were classified as depression (χ^2^[1] = 11.98, *p* < 0.001) or dementia (χ^2^[1] = 4.79, *p* = 0.029), whereas a higher percentage from the neurology clinic were classified as Normal Cognition (χ^2^[1] = 23.59, *p* < 0.001) or MCI (χ^2^[1] = 10.29, *p* = 0.001). A similar sized majority of patients at both sites were classified as either MCI or Dementia (primary care: 56.8% vs neurology specialty care: 59.4%; χ^2^[1] = 0.34; *p* = 0.558).

Supplementary analyses compared demographic characteristics and neuropsychological test scores of participants who fell into the four classification categories in each clinic setting (Tables S1 and S2). These analyses are for descriptive purposes only since test scores were used in determining group membership.

### 3.3 Recruitment into ADRD research

Similar percentages of those screened at the primary care (91.9%) or neurology (86.6%) clinic (χ^2^[1] = 3.31, *p* = 0.069) consented to be listed in the research registry and contacted about potential participation in ADRD research projects (Table 3). The percentages of those consenting were high (i.e., >80%) across all classifications (e.g., Normal, Depression, MCI, Dementia) in both clinic settings. Multivariate logistic regression using age, years of education, sex, language of testing, clinic setting, and final screening classification to predict those who did (*n* = 460) or did not (*n* = 60) consent to be enrolled in the research registry showed that age was the only significant predictor (*B* = −0.048, SE = 0.015, Wald = 9.95, *p* = 0.002, odds ratio = 0.953, 95% CI [.926, 0.982]), with younger individuals more likely to consent than older individuals.

**TABLE 3:** Number (and percentage) of screened patients from primary care versus neurology specialty care clinics who agreed to be listed in the Alzheimer's Disease Research Center (ADRC) research registry and contacted about research opportunities, and those from the registry who enrolled in any Alzheimer's disease and related dementias (ADRD) research study (including the ADRC longitudinal study). Those who enrolled in the ADRC longitudinal study are also shown separately.

	Primary care	Neurology specialty care
Total screened	(*n* = 185)	(*n* = 335)
**Agreed to contact and research registry**	170 (91.9%)	290 (86.6%)
Normal cognition	22/23 (95.7%)	67/77 (87.0%)
Depression	54/57 (94.7%)	53/59 (89.8%)
Mild cognitive impairment	25/27 (92.6%)	81/90 (90.0%)
Dementia	69/78 (88.5%)	89/109 (81.7%)
**Recruited into at least one ADRD study**	8/170 (4.7%)	23/290 (7.9%)
Normal cognition	0/23 (0%)	6/77 (7.8%)
depression	0/57 (0%)	3/59 (5.1%)
Mild cognitive impairment	1/27 (3.7%)	7/90 (7.8%)
Dementia	7/78 (9.0%)	7/109 (6.4%)
**Recruited into ADRC longitudinal study**	8/170 (4.7%)	13/290 (4.5%)

The 460 Latino participants who were screened and listed in the research registry supported 368 referrals to 45 different ADRD research studies across nine study types (e.g., interventions, neuroimaging, biomarker, neuropsychological) (see Table 4 and Table S3 for study‐specific data). Of the 460 Latino registry participants, 156 were referred to multiple studies, 224 were referred to only one study, and 80 were not referred. Most of the 368 referrals led to exclusion by the study team based on further record review or additional information gained after contact (56.5%), declining to participate after hearing details of the study and its requirements (18.2%), or failure of the study team to follow‐up on the referral (4.1%). However, 21.5% of all referrals led to successful enrollment in an ADRD research study.

**TABLE 4:** Number of participants referred from research registry to various types of Alzheimer's disease and related dementias (ADRD) studies. The number of participants referred, excluded for not meeting study inclusion/exclusion criteria (Excluded), that declined to participate (Declined), successfully enrolled (Enrolled), or not contacted after referral (No Contact) are shown. Note: The number of studies in each study type are shown in parentheses. Individuals could be referred to multiple studies across multiple study types.

Study type (number of studies)	Referred	Excluded	Declined	Enrolled	No Contact
Pharmacological intervention (8)	83	72	11	0	0
Behavioral intervention (2)	2	1	1	0	0
Neuropsychological (3)	3	2	0	1	0
Neuropsychological – Latino (5)	93	67	4	22	0
Behavioral (8)	110	37	40	26	7
Neuroimaging/electrophysiology (12)	39	17	2	17	3
Vascular assessment (2)	9	0	5	1	3
Cerebrospinal fluid (CSF) biomarker (3)	13	0	1	10	2
Induced pluripotent stem cells (2)	17	12	3	2	0
**Totals**	368	208	67	79	15
**Percentage of total referrals (*n* = 368)**	—	56.5%	18.2%	21.5%	4.1%

Thirty‐one individuals participated in at least one ADRD research project (6.7% of 460) with no difference in the percentage from each clinic (χ^2^[1] = 0.4, *p* = 0.530) (Table 3). The majority had been classified as MCI or Dementia at screening (*N* = 22; 71.0%). Those who enrolled in ADRD studies were similar to all participants who agreed to be listed in the registry in average age (enrolled in ADRD studies: 72.90 ± 10.17, registry: 72.59 ± 10.05), years of education (enrolled in ADRD studies: 8.06 ± 3.19, registry: 6.93 ± 4.25), and MMSE (enrolled in ADRD studies: 23.97 ± 3.50, registry: 23.76 ± 4.61) and in percentage female (enrolled in ADRD studies: 54.8%, registry: 62.0%), tested in Spanish (enrolled in ADRD in studies: 96.8%, registry: 93.7%), and from the neurology specialty clinic (enrolled in ADRD studies: 74.2%, registry: 63.0%). While the number of screened individuals who subsequently participated in ADRD research (*N* = 31) was comparatively small, many of them participated in multiple projects (average number of projects = 3.13, SD = 2.29, range 1 to 9), accounting for 79 distinct enrollments across 15 unique ADRD studies.

## 4 DISCUSSION

Embedding opportunities to engage in ADRD research within community‐based medical care of older Latino adults resulted in a high rate of participation. Essentially all (>99%) older Latino patients with concerns about their cognition agreed to allow cognitive screening data to be used for research purposes, and more than 85% agreed to being listed in a research registry and consented to be contacted about future ADRD research opportunities. This high level of agreement demonstrates the effectiveness of community‐based participatory research principles^15^ and shows the power of placing the discussion of ADRD research within the context of trusted community‐based medical care. Our model cognitive screening program allowed us to develop a presence in the community, provided a needed and helpful service, and gave us the opportunity to educate the community about the value of research. Our success in using this approach to generate interest in research and populate an ADRD research registry with older Latino adults at risk of cognitive decline (i.e., 460 of 520 screened, or 88.5%) was equally or more effective than other recruitment strategies such as earned media, community education (e.g., 64%),^24^ or partnering with community organizations (e.g., 52.6%)^25^ to identify interested individuals from underrepresented groups.^26^, ^27^, ^28^, ^29^ However, enrollment of those in the research registry into an ADRD research study was modest (approximately 7%), for reasons described below.

The willingness of older Latino adults in two separate clinic‐based populations to allow their cognitive screening data to be used for research purposes supports the feasibility of pragmatic trials in ADRD.^30^ Pragmatic trials are designed to examine the effectiveness of a health intervention in real‐world clinical practice, generally employing methods that are part of routine care.^31^ Our results show that older Latino adults with subjective or objective cognitive impairment are highly likely to allow data collected as part of routine clinical care to be used in research (e.g., as treatment outcome measures) and are not averse to clinical research, per se. A pragmatic approach could greatly reduce barriers and health disparities that disproportionately prevent this underrepresented population from participating in ADRD clinical trials.^32^, ^33^, ^34^

Similarly high numbers of patients from each clinic agreed to allow screening data to be used for research purposes (>99% at each site) and to participate in the research registry (92% primary care, 87% neurology specialty care). Clinic setting, sex, education, language of testing, and screening classification were not associated with the decision to participate; however, older patients were less likely to agree. Patients screened in the neurology clinic were older and had more years of formal education than those from the primary care clinic, and a lower proportion were women, differences that could reflect less access to specialty neurology care for women or those with fewer resources.^35^ Groups did not differ in the proportion with concomitant medical problems that might contribute to cognitive decline (e.g., traumatic brain injury, stroke, clinical depression), but those screened at the neurology clinic had lower GDS scores and were more likely to be taking antidepressant medications, perhaps because treatment for depressed mood is initiated at the primary care level before referral for specialty neurology assessment of cognitive decline, or patients were referred to psychiatry specialty care for further evaluation and treatment. Overall, more than 22% of those screened were classified as Depression (i.e., above cut‐offs for depressive symptoms on the GDS without significant cognitive impairment), consistent with prevalence estimates for depression in Latino older adults.^36^

Patients screened at the primary care clinic had more subjective cognitive complaints than those screened at the neurology clinic and scored worse on MMSE and other objective neuropsychological tests, even after consideration of age and education. This difference was reflected in screening outcomes with a higher proportion of primary care than neurology clinic patients categorized as Dementia and a lower proportion categorized as Normal Cognition. This suggests that primary care retained management of patients with more severe and obvious cognitive impairment but referred those with more subtle cognitive decline (e.g., many who end up being treated as having normal cognition by screening) to neurology specialty care for more detailed evaluation. Interestingly, patients screened in the neurology clinic were more likely than those screened in primary care to already be on a cholinesterase inhibitor medication at the time of screening, suggesting that treatment for cognitive impairment may often begin at the primary care level before referral to neurology specialty care for further evaluation and ongoing treatment monitoring.^37^

Our results have implications for choosing where clinic‐based cognitive screening is deployed as a recruitment strategy to increase Latino participation in ADRD research. If the targeted cohort is patients with dementia or those naïve to antidepressant or cholinesterase inhibitor treatment, then the primary care setting may be preferred. On the other hand, if the targeted cohort is patients with milder stages of disease or normal cognition with subjective cognitive complaints not due to depressed mood, then the neurology specialty care setting may be favored. Otherwise, the two settings are very similar in patient characteristics and likelihood that Latino older adults will agree to participate.

Approximately 7% of the older Latino adults who agreed to be listed in the research registry eventually enrolled in at least one of 45 actively recruiting ADRD studies. This percentage is lower than at least one study of non‐Latino Whites that reported that 17.3% of patients who received clinic‐based cognitive screening were recruited into an ADRD clinical trial.^38^ Barriers to successful enrollment from the Latino cohort may account for the lower percentage. Previously identified barriers from the researchers’ side include not having recruitment materials and study information available in Spanish,^6^ not having translated materials and consent forms,^14^ and not having bilingual staff to perform cognitive testing and data collection in Spanish. These constraints were present in most of our affiliated studies, which excluded those who did not fluently speak English. In addition, many recruiting studies had restrictive inclusion (e.g., small MMSE range) or exclusion (e.g., no concomitant medical issues) criteria that limited enrollment from this cohort. Barriers from the participants’ side include concern about physical distance from research facilities (e.g., neuroimaging center), transportation limitations, financial burden, and anxiety concerning certain research procedures (e.g., lumbar puncture, medication).^11^ Many of the recruiting studies were subject to these barriers and did not have the capacity to try to overcome them due to study design, study timeline, or other resource limitations. In some cases, researchers had a preconceived notion of a lack of interest in research participation in the Latino community and did not try to address barriers. Contrary to this perception, our results show that there exist an interest and a willingness to participate in ADRD research if barriers can be overcome, perhaps by using community‐based participatory and pragmatic approaches. Indeed, in our cohort, older Latino adults successfully recruited into an ADRD research study were highly motivated and participated in more than three (on average) of the 15 unique research projects that successfully enrolled at least one registry participant.

Several limitations in this study should be noted. First, the screening procedures we employed were developed and validated in a largely non‐Latino White cohort with a relatively high level of education.^16^ We did not have independent validation of the accuracy of our screening classifications in the older Latino cohort. However, all test procedures had been used and validated for the detection of cognitive impairment in Spanish‐speaking older Latino adults in the ADRC longitudinal observational study.^13^, ^22^, ^39^ Future studies should ensure the validity of these procedures in the screening setting and determine how they can be effectively integrated with newly emerging plasma‐based biomarkers for AD.^40^ Second, referrals for screening at the primary care clinic were made by 14 different practitioners, whereas those referred at the neurology specialty care clinic were almost exclusively from one neurologist. Thus, results from the neurology specialty care clinic may be less generalizable than those from the primary care clinic. Finally, it should be noted that our screening program may not be ideal for recruiting individuals with normal cognition for AD prevention studies since even those who screened as normal cognition had subjective cognitive decline based on a complaint of memory or cognitive decline from the patient, a family member, or a physician suspecting cognitive decline. Other methods to boost recruitment of Latino individuals for prevention studies, such as education and engagement through Latino community health care workers (i.e., “Promotoras”)^41^ or free community memory screening,^42^ may be more effective, particularly when combined with methods to reduce barriers such as improving social media presence with Spanish language content, establishing research facilities in the Latino community in partnership with trusted community partners, offering compensation for transportation and time spent, and relaxing requirements for difficult research procedures (e.g., lumbar puncture) as much as possible.^11^ Despite these limitations, our screening procedures provided a needed clinical service to the community while facilitating participation of the underrepresented older Latino population in ADRD research.

## CONFLICTS OF INTEREST STATEMENT

Dr. David Salmon was a paid consultant for Aptinyx and Biogen. Dr. Doug Galasko is a paid consultant for Biogen, Fujirebio, Cognition Therapeutics, Amprion, vTv Pharmaceuticals, and GE Healthcare. There are no other financial disclosures to report relevant to this manuscript. Author disclosures are available in the supporting information.

## CONSENT STATEMENT

The research protocol was approved by the UCSD Institutional Review Board in accordance with the Helsinki Declaration. Written informed consent was obtained from all patients, consistent with California State law, to utilize deidentified data for research purposes, include their name and contact information in a research registry, and permission to contact them about future ADRD research opportunities.

## Supporting information



Supporting Information

Supporting Information

Supporting Information

Supporting Information
