# Supplementary material for: A cognitive screening program in community‐based medical clinics to facilitate Latino participation in Alzheimer's disease research
Source: Alzheimers Dement. 2026 Jan 22;22(1):e71132. doi: 10.1002/alz.71132 (PMC12826067; doi:10.1002/alz.71132)
Supplement: Supplementary file 1 — Supporting Information [file ALZ-22-e71132-s002.docx]

Supplementary Table 1

Mean (s.d.) age, education, grade equivalency, and scores on the Subjective Cognitive Decline (SCD), Geriatric Depression Scale, Mini-Mental State Exam, and cognitive screening tests of **primary care clinic** patients as a function of cognitive screening classification group.

Normal Cognition Depression MCI Dementia One-Way ANOVA

(n=23) (n=57) (n=27) (n=78)

Age 64.0 (7.6) 63.5 (8.3) 63.9 (7.4) 72.2 (11.0) ^a,b,c^ F(3,181)=12.38; p<.001

Education (years) 7.0 (4.0) 6.1 (3.5) 6.7 (4.3) 4.9 (3.3) ^a,b,c^ F(3,180)= 3.22; p=.024

Grade Equivalency 13.8 (5.2) 11.2 (5.4) 10.5 (5.3) ^a^ 10.0 (5.4) ^a^ F(3,158)= 2.93; p=.035

Sex (M/F) 6/17 15/42 8/19 29/49

Geriatric Depression Scale (GDS) 3.2 (1.4) 9.5 (3.1) ^a^ 4.4 (3.7) ^b^ 6.3 (4.8) ^a,b,c^ F(3,147)=16.24; p<.001

SCD Total Score 3.3 (1.7) 3.8 (1.2) 3.3 (1.6) 3.2 (1.5) ^b^ F(3,181)= 2.14; p=.097

Mini-Mental State Exam (MMSE) 27.5 (2.3) 24.2 (4.8) ^a^ 23.6 (3.2) ^a^ 19.2 (4.9) ^a,b,c^ F(3,180)=27.13; p<.001

Logical Memory Test (Story A)

Immediate 12.3 (2.9) 8.4 (3.5) ^a^ 8.3 (3.6) ^a^ 3.7 (2.7) ^a,b,c^ F(3,179)=54.68; p<.001

Delayed 10.5 (3.0) 7.1 (3.6) ^a^ 6.2 (3.8) ^a^ 2.1 (2.1) ^a,b,c^ F(3,176)=59.23; p<.001

Percent Savings (%) 83.9 (11.6) 81.7 (26.5) 71.7 (28.2) 43.8 (41.8) ^a,b,c^ F(3,173)=18.00; p<.001

Trail-Making Test A (sec.) 65.0 (24.5) 77.6 (32.7) 85.9 (41.5) ^a^ 106.0 (40.2) ^a,b,c^ F(3,156)= 9.33; p<.001

Trail-Making Test B (sec.) 197.2 (88.5) 201.1 (82.9) 226.2 (75.5) 285.0 (35.0) ^a,b,c^ F(3,110)=10.22; p<.001

CERAD Word List Learning

Trials 1-3 Total 17.7 (3.7) 16.2 (3.5) 13.1 (3.6) ^a,b^ 9.8 (3.8) ^a,b,c^ F(3,177)=44.73; p<.001

Delayed Recall 6.3 (1.4) 5.9 (1.4) 4.3 (2.1) ^a,b^ 1.7 (1.7) ^a,b,c^ F(3,177)=89.33; p<.001

Correct Recognition 18.9 (2.0) 18.3 (1.8) 17.0 (2.9) ^a,b^ 15.6 (2.9) ^a,b,c^ F(3,176)=17.71; p<.001

Clock Drawing Test 2.3 (0.7) 2.0 (0.9) 2.0 (0.8) 1.4 (0.7) ^a,b,c^ F(3,174)=10.58; p<.001

Category Fluency (“Animals”) 17.2 (4.9) 14.0 (4.2) ^a^ 14.4 (3.6) ^a^ 11.2 (4.1) ^a,b,c^ F(3,176)=14.33; p<.001

Note: MCI=Mild Cognitive Impairment; post-hoc group comparison with Least Significant Difference (LSD test, significance set at p < .05.

^a^ different from Normal Cognition

^b^ different from Depression

^c^ different from MCI
