# Supplementary material for: A cognitive screening program in community‐based medical clinics to facilitate Latino participation in Alzheimer's disease research
Source: Alzheimers Dement. 2026 Jan 22;22(1):e71132. doi: 10.1002/alz.71132 (PMC12826067; doi:10.1002/alz.71132)
Supplement: Supplementary file 2 — Supporting Information [file ALZ-22-e71132-s003.docx]

Supplementary Table 2

Mean (s.d.) age, education, grade equivalency, and scores on the Subjective Cognitive Decline (SCD) scale, Geriatric Depression Scale, Mini-Mental State Exam, and cognitive screening tests of **neurology specialty care clinic** patients as a function of cognitive screening classification group.

Normal Cognition Depression MCI Dementia One-Way ANOVA

(n=77) (n=59) (n=90) (n=109)

Age 73.4 (7.2) 70.0 (9.4) ^a^ 75.3 (8.8) ^b^ 78.0 (7.9) ^a,b,c^ F(3,331)=13.34; p<.001

Education (years) 8.2 (4.8) 7.2 (4.2) 7.2 (4.7) 7.0 (4.4) F(3,330)= 1.24; p=.295

Grade Equivalency 14.2 (4.9) 13.0 (5.1) 11.1 (5.2) ^a,b^ 10.1 (4.9) ^a,b,c^ F(3,292)=10.18; p<.001

Sex (M/F) 29/48 17/42 47/43 46/63

Geriatric Depression Scale (GDS) 2.1 (3.1) 7.7 (2.5) ^a^ 2.4 (2.3) ^b^ 3.7 (3.4) ^a,b,c^ F(3,328)=50.55; p<.001

SCD Total Score 1.9 (1.5) 3.1 (1.6) ^a^ 2.0 (1.5) ^b^ 2.4 (1.8) ^b^ F(3,330)= 7.29; p<.001

Mini-Mental State Exam (MMSE) 27.2 (2.4) 25.3 (3.0) ^a^ 24.8 (3.4) ^a^ 21.7 (3.9) ^a,b,c^ F(3,331)=44.41; p<.001

Logical Memory Test (Story A)

Immediate 11.3 (3.1) 8.8 (3.5) ^a^ 7.9 (3.2) ^a^ 4.7 (3.1) ^a,b,c^ F(3,330)=66.96; p<.001

Delayed 10.1 (3.3) 6.9 (3.6) ^a^ 5.3 (3.3) ^a,b^ 2.4 (2.4) ^a,b,c^ F(3,330)=95.89; p<.001

Percent Savings (%) 87.9 (20.5) 78.0 (33.0) 65.1 (29.2) ^a^ 53.1 (83.7) ^a,b^ F(3,328)= 7.31; p<.001

Trail-Making Test A (sec.) 61.8 (36.1) 70.2 (37.9) 77.7 (36.2) ^a^ 101.7 (38.2) ^a,b,c^ F(3,320)=18.92; p<.001

Trail-Making Test B (sec.) 184.9 (86.0) 218.6 (87.8) ^a^ 246.2 (73.4) ^a^ 287.9 (40.6) ^a,b,c^ F(3,226)=19.19; p<.001

CERAD Word List Learning

Trials 1-3 Total 17.4 (3.2) 15.6 (3.6) ^a^ 14.2 (3.3) ^a,b^ 10.8 (3.3) ^a,b,c^ F(3,324)=64.89; p<.001

Delayed Recall 6.3 (1.5) 5.3 (2.1) ^a^ 4.1 (2.1) ^a,b^ 2.0 (1.6) ^a,b,c^ F(3,323)=91.63; p<.001

Correct Recognition 19.4 (0.9) 19.0 (1.5) 18.1 (2.1) ^a,b^ 16.1 (2.8) ^a,b,c^ F(3,322)=44.71; p<.001

Clock Drawing Test 2.2 (0.7) 2.2 (0.8) 2.0 (0.7) ^a^ 1.6 (0.7) ^a,b,c^ F(3,326)=17.36; p<.001

Category Fluency (“Animals”) 17.0 (5.3) 14.9 (4.3) ^a^ 14.3 (3.6) ^a^ 11.3 (3.9) ^a,b,c^ F(3,326)=27.07; p<.001

Note: MCI=Mild Cognitive Impairment; post-hoc group comparison with Least Significant Difference (LSD test, significance set at p < .05.

^a^ different from Normal Cognition

^b^ different from Depression

^c^ different from MCI
