# Supplementary material for: A cognitive screening program in community‐based medical clinics to facilitate Latino participation in Alzheimer's disease research
Source: Alzheimers Dement. 2026 Jan 22;22(1):e71132. doi: 10.1002/alz.71132 (PMC12826067; doi:10.1002/alz.71132)
Supplement: Supplementary file 3 — Supporting Information [file ALZ-22-e71132-s004.docx]

Supplementary Table 3

The number of Latino participants from the Cognitive Screening Program who were referred from the ADRC Research Registry to various Alzheimer’s Disease and Related Disorders (ADRD) research studies. The number referred to each study, referred who did not meet study inclusion/exclusion criteria (Excluded), declined to participate (Declined), successfully enrolled (Enrolled), or were not contacted after referral (No Contact) are shown. Note that an individual participant could be referred to multiple studies in multiple study types.

| **Affiliated Projects Using ADRC Research Registry** | **Study Type** | **Referred** | **Excluded** | **Declined** | **Enrolled** | **No Contact** |
| --- | --- | --- | --- | --- | --- | --- |
| A4 Clinical Trial | Pharmacological Intervention | 1 | 1 | 0 | 0 | 0 |
| Accera - AC-1204 Long-Term Efficacy | Pharmacological Intervention | 1 | 1 | 0 | 0 | 0 |
| BHV4157 (T2) Trial-Biohaven | Pharmacological Intervention | 61 | 60 | 1 | 0 | 0 |
| Biomarker Predictors of Memantine Sensitivity in Alzheimer's Disease | Pharmacological Intervention | 3 | 2 | 1 | 0 | 0 |
| Discover (Posiphen) Trial | Pharmacological Intervention | 1 | 1 | 0 | 0 | 0 |
| Lundbeck-Lu AE 58054 Clinical Trial | Pharmacological Intervention | 1 | 1 | 0 | 0 | 0 |
| Organic Triphala and VSL#3 Probiotics on Stool Microbiome Profiles & Inflammation | Pharmacological Intervention | 8 | 0 | 8 | 0 | 0 |
| Salsalate AD Clinical Trial | Pharmacological Intervention | 7 | 6 | 1 | 0 | 0 |
| Combined Behavioral Interventions on Cognitive Outcomes in MCI | Behavioral Intervention | 1 | 0 | 1 | 0 | 0 |
| Novel Mobile Health Exercise Intervention in Aging: Brain Perfusion and Cognition | Behavioral Intervention | 1 | 1 | 0 | 0 | 0 |
| Visual Perception in Dementia with Lewy Bodies | Neuropsychological | 1 | 1 | 0 | 0 | 0 |
| Pattern Separation in MCI | Neuropsychological | 1 | 0 | 0 | 1 | 0 |
| Pupillary Responses as a Risk and Staging Biomarker of Preclinical AD | Neuropsychological | 1 | 1 | 0 | 0 | 0 |
| Advancing Reliable Measurement in AD and Cognitive Aging (ARMADA Toolbox) | Neuropsychological-Hispanic | 57 | 51 | 4 | 2 | 0 |
| Bilingual Alzheimer's Disease | Neuropsychological-Hispanic | 27 | 11 | 0 | 16 | 0 |
| Bilingual Protocol - GALIDISS | Neuropsychological-Hispanic | 4 | 0 | 0 | 4 | 0 |
| Bilingualism, Aging and AD | Neuropsychological-Hispanic | 3 | 3 | 0 | 0 | 0 |
| MINT SPRINT Validation Study | Neuropsychological-Hispanic | 2 | 2 | 0 | 0 | 0 |
| Advanced Care Planning-Caregiver Survey | Behavioral | 8 | 0 | 0 | 8 | 0 |
| Alzheimer's Caregiver Coping: Mental and Physical Health | Behavioral | 11 | 11 | 0 | 0 | 0 |
| Caregiver Pleasant Events Project | Behavioral | 11 | 11 | 0 | 0 | 0 |
| Cognitive Decline in Hispanic vs. Non-Hispanic Spousal Alzheimer's Caregivers | Behavioral | 3 | 0 | 3 | 0 | 0 |
| Quality of Life Programs | Behavioral | 7 | 0 | 0 | 0 | 7 |
| Subjective Cognitive Decline (SCD) in Older Latinos | Behavioral | 2 | 0 | 0 | 2 | 0 |
| Subjective Cognitive Decline in Hispanics (RCMAR Project) | Behavioral | 15 | 1 | 0 | 14 | 0 |
| Successful Aging Among Latinos in the U.S.-Salus Study | Behavioral | 53 | 14 | 37 | 2 | 0 |
| Cognitive and Brain Changes in Preclinical Alzheimer's Disease | Neuroimaging | 1 | 1 | 0 | 0 | 0 |
| DVCID-Vascular Cognitive Impairment | Neuroimaging | 2 | 0 | 0 | 2 | 0 |
| Functional and Structural Neuroanatomy of Past Remembrance | Neuroimaging | 2 | 2 | 0 | 0 | 0 |
| Locus Coeruleus in Preclinical AD | Neuroimaging | 1 | 1 | 0 | 0 | 0 |
| Microstructural Biomarkers of MCI and AD | Neuroimaging | 9 | 9 | 0 | 0 | 0 |
| Neuroimaging and Vascular Correlates of MCI Subtypes | Neuroimaging | 1 | 0 | 1 | 0 | 0 |
| Novel Locus Coeruleus Marker of Inflammation and Risk of AD | Neuroimaging | 3 | 3 | 0 | 0 | 0 |
| Quantitative Imaging in Dementia with Lewy Bodies | Neuroimaging | 1 | 0 | 0 | 1 | 0 |
| SCAN PET Amyloid Imaging | Neuroimaging | 2 | 0 | 1 | 1 | 0 |
| Volumetric MRI in AD | Neuroimaging | 13 | 0 | 0 | 13 | 0 |
| Assessing Neural Networks Using EEG | Electrophysiology | 1 | 1 | 0 | 0 | 0 |
| Integrated Neurocognitive and Sleep-Behavior Profiler for Dementia Classification | Electrophysiology | 3 | 0 | 0 | 0 | 3 |
| Cerebrovascular Function Assessment and Arterial Stiffness Assessment | Vascular Assessment | 2 | 0 | 2 | 0 | 0 |
| Pulse Wave Velocity in AD | Vascular Assessment | 7 | 0 | 3 | 1 | 3 |
| APOE Multi-Center CSF Biomarker Study | CSF Biomarker | 1 | 0 | 1 | 0 | 0 |
| CSF AD Biomarker | CSF Biomarker | 10 | 0 | 0 | 10 | 0 |
| Extracellular RNA as an AD Biomarker | CSF Biomarker | 2 | 0 | 0 | 0 | 2 |
| Induced Pluripotent Stem Cell Initiative | Induced Pluripotent Stem Cell | 12 | 7 | 3 | 2 | 0 |
| Stem Cell Models of AD | Induced Pluripotent Stem Cell | 5 | 5 | 0 | 0 | 0 |
| Totals |  | 376 | 210 | 69 | 80 | 17 |
